# Differential Epitope Mapping by STD NMR Spectroscopy To Reveal the Nature of Protein–Ligand Contacts

**DOI:** 10.1002/anie.201707682

**Published:** 2017-10-23

**Authors:** Serena Monaco, Louise E. Tailford, Nathalie Juge, Jesus Angulo

**Affiliations:** ^1^ School of Pharmacy University of East Anglia Norwich Research Park Norwich UK; ^2^ The Gut Health And Food Safety Institute Strategic Program Quadram Institute of Bioscience NR47UA Norwich Research Park Norwich UK

**Keywords:** epitope mapping, fragment-based drug design, NMR spectroscopy, pharmacophores, protein–ligand binding

## Abstract

Saturation transfer difference (STD) NMR spectroscopy is extensively used to obtain epitope maps of ligands binding to protein receptors, thereby revealing structural details of the interaction, which is key to direct lead optimization efforts in drug discovery. However, it does not give information about the nature of the amino acids surrounding the ligand in the binding pocket. Herein, we report the development of the novel method differential epitope mapping by STD NMR (DEEP‐STD NMR) for identifying the type of protein residues contacting the ligand. The method produces differential epitope maps through 1) differential frequency STD NMR and/or 2) differential solvent (D_2_O/H_2_O) STD NMR experiments. The two approaches provide different complementary information on the binding pocket. We demonstrate that DEEP‐STD NMR can be used to readily obtain pharmacophore information on the protein. Furthermore, if the 3D structure of the protein is known, this information also helps in orienting the ligand in the binding pocket.

Saturation transfer difference (STD) NMR spectroscopy is a powerful NMR technique for ligand screening and gaining quantitative structural information from biologically relevant protein–ligand complexes.^1^ The approach is appropriate for small‐molecule binders of medium‐weak affinity (high nm to low mm), there is no upper limit for protein size,^2^ and labelling is not required. The technique is highly versatile and popular in the context of hit identification in drug discovery.^3^

STD NMR is based on the generation of saturation on a selected group of protein protons through selective “saturating” irradiation. For large proteins, spin diffusion spreads the saturation over the entire macromolecule, ultimately leading to intermolecular NOEs with protons of the ligand in the binding pocket. Mapping the STD NMR intensities on the ligand structure allows identification of “all” the ligand contacts with the protein in the complex (group epitope mapping).1b However, it fails to provide information about the ligand location/orientation in the bound state. For this, competitive STD NMR experiments with “spy” ligands of known binding location must be carried out.^4^

Importantly, spin diffusion does not always ensure homogeneous saturation over the receptor, and differences in ligand epitope maps can be observed if STDs are acquired at different saturating frequencies.^5^, ^6^ This is prominent in the case of nucleic acids, where these differences are exploited to distinguish between major‐ and minor‐groove binders.^6^ For proteins, no methods have been described to exploit these differences to gain additional structural information. Although for proteins, these differences are typically small, they can be magnified and mapped into the ligand. Notably, ligand protons close to directly irradiated protein protons will show a relative increase in STDs compared to conditions where they are not directly irradiated (Figure 1 a). Since the frequencies of irradiation can be chosen, we can select what types of protein protons will be “directly irradiated”, so that the differences will highlight parts of the ligand contacting those types of protein residues in the bound state (Figure 1 a).

**Figure 1:**
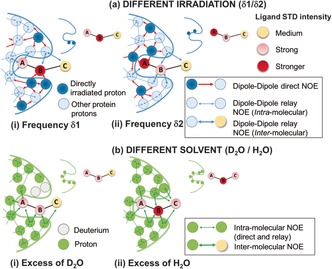
A cartoon representing the two implementations of the DEEP‐STD NMR method. a) Different irradiation frequencies: ligand protons receive larger saturation if the protein protons in their proximity are “directly irradiated” instead of “relayed‐NOE” saturated. STD NMR is carried out with selective irradiation (δ1) on protein protons close to ligand proton B (i) and with selective irradiation (δ2) on protein protons close to ligand proton A (ii). The distinct binding epitopes of the ligand are sketched in the free state. b) Different solvent composition: ligand protons close to slowly exchanging protein exchangeable protons receive less saturation if the latter are exchanged to deuterium (in D_2_O) instead of a proton (in H_2_O). STD NMR experiments are thus carried out in D_2_O (i) and H_2_O (ii).

Another source of minor differences in epitope maps is the solvent. In D_2_O, the polar side chains in the binding pocket have their exchangeable protons replaced by ^2^H, which is inefficient for transferring saturation.^7^ In H_2_O, these protons can contribute to an additional transfer of saturation compared to D_2_O.^8^ This process depends on their exchange rate with bulk water, with slowly exchanging polar protons being expected to produce the largest variations.^8^ Therefore, ligand protons contacting these polar residues will show a relative increase in STDs compared to the binding epitopes in D_2_O (Figure 1 b).

In this work, we proposed to exploit these epitope differences to get information on the types of protein protons (aromatic, polar, or apolar) the ligand is contacting, thereby allowing assessment of the pharmacophore of the protein. To detect and quantify these differences, we have designed a simple method consisting of running pairs of STD NMR experiments under two experimental conditions (Figure 1) and quantifying the differences in relative STDs. Each pair consists of experiment‐1 (*exp1*) and experiment‐2 (*exp2*), performed under two conditions, that is, two different frequencies or two different solvents (D_2_O or H_2_O). The result provides a “Differential Epitope Map” of the ligand, which is a map of epitope differences from each pair of STD NMR experiments. We call the method differential epitope mapping STD NMR spectroscopy (DEEP‐STD NMR).

The differential epitope map of a ligand under two experimental conditions (*exp1* and *exp2*) is determined by the DEEP‐STD factor (ΔSTD_*i*_) for each proton *i* as:

(1) $${\Delta STD}_{i}=\;\frac{{STD\;}_{exp1,i}}{{STD\;}_{exp2,i}}-\;\frac{1}{n}\sum_{i}^{n}\left(\frac{{STD\;}_{exp1,i}}{{STD\;}_{exp2,i}}\right)$$

To obtain a consistent scale of ΔSTD_*i*_ factors, *exp1* must be the experiment showing larger total ligand saturation. The term $\left(\left(1/n\right)\sum_{i}^{n}\left({STD\;}_{exp1,\;i}/{STD\;}_{exp2,\;i}\right)\right)$ accounts for intrinsic differences in saturation levels under different conditions. Ligand protons not affected by the changes in experimental conditions should show low ΔSTD_*i*_ factors, ideally close to 0. The differential epitope map of the ligand is obtained by mapping the ΔSTDs onto its structure. Notably, since ΔSTDs reflect contacts to specific types of amino acids, if the 3D structure of the protein is known, the method can also reveal the orientation of the ligand in the binding pocket.

As a proof of principle, we studied two biologically relevant protein–ligand complexes for which high‐resolution X‐ray structures are available: 1) 2,7‐anhydro‐Neu5Ac with *Rg*NanH‐GH33, the catalytic domain (belonging to glycoside family GH33) of the intramolecular trans‐sialidase from human the gut symbiont *Ruminococcus gnavus,* 9 and 2) 3‐nitrophenyl‐α‐d‐galactopyranoside (3NPG) with Cholera toxin subunit B (CTB).^10^ Understanding the binding of 2,7‐anhydro‐Neu5Ac by sialidases is important to unveil mechanisms of gut microbiota adaptation.^9^, ^11^ 3NPG is a well‐known inhibitor of CTB and is frequently chosen as a reference for the design of novel inhibitors.

We first tested the method by analyzing the effect of different irradiation frequencies on the binding of 2,7‐anhydro‐Neu5Ac to *Rg*NanH‐GH33. For DEEP‐STD NMR, the selection of frequencies to irradiate different types of protein protons can be based on the spectral properties of the protein (if chemical shifts are assigned), NMR databases (e.g., BMRB^12^), or predictions using a 3D model of the protein.^13^ We ran two STD NMR experiments irradiating (0.5 s) at 0.60 ppm (*exp1*) and 6.55 ppm (*exp2*). These frequencies are known to be centered in the aliphatic and aromatic protein spectral regions, respectively.^12^

Several ΔSTD factors were observed (Figure 2 a), confirming changes in the ligand binding epitope map under the two different irradiations. The ΔSTD factors (0.60/6.55 ppm) are shown in Figure 2 a.

**Figure 2:**
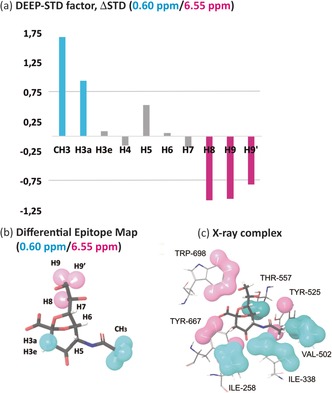
Differential Epitope Mapping (0.6/6.55 ppm) of 2,7‐anhydro‐Neu5Ac in complex with *Rg*NanH‐GH33. a) ΔSTD histogram: positive ΔSTDs (above the limit of +0.75) after aliphatic irradiation (0.6 ppm) are shown in cyan, and negative ΔSTDs (below −0.75) after aromatic irradiation (6.55 ppm) in magenta. b) DEEP‐STD map of the ligand. Cyan surfaces highlight ligand contacts with aliphatic side chains; magenta surfaces show contacts with aromatic side chains. c) Crystal structure of the complex (PDB ID: 4X4A).^9^ Protons were added using Schrodinger software.^14^ Protein protons are colored as a function of their NMR frequencies: those directly irradiated at 0.6 ppm (aliphatic residues) in cyan and those directly irradiated at 6.55 ppm (aromatic residues) in magenta (Table S1). Comparison of (b) and (c) highlights the excellent match of the differential epitope map of the ligand with the distribution of aliphatic and aromatic residues in the binding pocket.

Positive ΔSTD factors report relative STD increases with irradiation at 0.60 ppm (aliphatic residues), whereas negative ones indicate increases when irradiating at 6.55 ppm (aromatic residues). The resulting differential epitope map is shown in Figure 2 b. The results show that different protons of the ligand occupy distinct areas of the *Rg*NanH‐GH33 binding pocket lined by either aliphatic or aromatic residues. The positive ΔSTD factors for CH_3_ and H3a suggest vicinity to aliphatic side chains, whereas the negative ΔSTDs for H8, H9 and H9′ suggest vicinity to aromatic protons.

The DEEP‐STD NMR results are in excellent agreement with the published crystal structure of the complex between 2,7‐anhydro‐sialic acid and *Rg*NanH‐GH33 (Figure 2 c),^9^ where the ligand sits between aliphatic (I258, I338, and V502) and opposite aromatic (Y667 and W698) patches. The ligand protons CH_3_ and H3a point towards the aliphatic residues, while H8, H9, and H9′ are projected towards the aromatic side chains. Protons H3e, H4, H5, H6, and H7 sit in between these two regions in agreement with their negligible ΔSTD factors (Figure 2 a). These results confirm that it is possible to identify the nature of the ligand–receptor contacts by means of differential protein irradiation.

We next studied the same protein–ligand system under two different solvent conditions: D_2_O (*exp1*) or H_2_O (*exp2*). The irradiation frequency was set at 0.6 ppm. In H_2_O, the large pool of solvent protons acts as a magnetization sink,^15^ resulting in a global saturation lower than in D_2_O. For this reason, we set *exp1* in Equation 1 to be the experiment in D_2_O. Thus, negative ΔSTD factors correspond to ligand protons with reduced relative STDs in D_2_O, that is, adjacent to slow exchanging polar residues in the bound state. We recorded negative ΔSTD factors from differential D_2_O/H_2_O experiments at protons H3a, H3e, H9, and H9′ (Figure 3 a). The resulting differential epitope map is shown in Figure 3 b, and portrays the areas of the ligand contacting polar residues. Here again, the DEEP‐STD NMR results were in line with the crystal structure (Figure 3 c), where the ligand protons H3a, H3e, H9, and H9′ point towards a highly polar patch in the *Rg*NanH‐GH33 binding pocket (R257, R276, R575, and R637). This is in excellent agreement with the known slow exchanging behavior of η protons of arginine residues in H_2_O.^8^ Interestingly, the ligand methyl group showed a positive ΔSTD factor (Figure 3 a), which is explained by the presence of a fast exchanging hydroxy group (Y525) close to the methyl groups of V502 (Figure 2 c and Figure S1 in the Supporting Information).^8^

**Figure 3:**
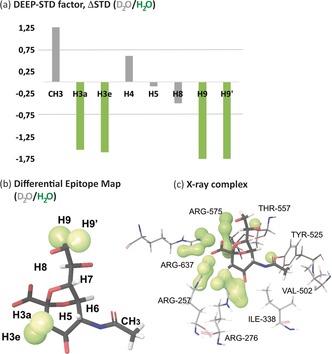
Differential Epitope Mapping (D_2_O/H_2_O) of 2,7‐anhydro‐Neu5Ac in complex with *Rg*NanH‐GH33. a) ΔSTD histogram: protons with an ΔSTD factor <−0.75 are shown in green. Protons H6 and H7 were excluded from the analysis due to their proximity to the water peak and the use of solvent suppression. b) DEEP‐STD map of the ligand. Green surfaces indicate ligand contacts with protein side chains carrying slowly exchanging protons. c) Crystal structure of the complex (PDB ID: 4X4A).^9^ Protons were added using Schrodinger software.^14^ The slowly exchangeable protons in the binding pocket are depicted with green surfaces.

Further, we applied this method to a complex of CTB, a larger receptor (65 kDa), with 3NPG.^10^ Remarkably, the ligand contains an aromatic moiety, which precludes protein irradiation in this spectral region. However, in DEEP‐STD NMR, it is possible to select other groups of protein protons for irradiation, providing that they are in spectral regions devoid of ligand signals. For CTB, we targeted protein resonances at 2.25 ppm. We predicted the chemical shifts of the protons of CTB within 4 Å of the ligand in the X‐ray structure, and the results indicated the E51 and Q56 protons as the ones likely to be directly irradiated (Figure 4 c, and Table S1 in the Supporting Information).

**Figure 4:**
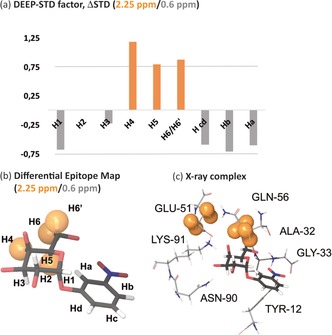
Differential Epitope Mapping (2.25/0.6 ppm) of 3‐nitrophenyl‐α‐d‐galactopyranoside (3NPG) in complex with Cholera toxin subunit B (CTB). a) ΔSTD histogram: protons with positive ΔSTDs (above the limit of +0.75) after irradiation at 2.25 ppm are shown in orange. b) DEEP‐STD map of the ligand. Orange surfaces indicate ligand contacts with protein side chains directly irradiated at 2.25 ppm. The ligand polar protons have been omitted. c) Crystal structure of the complex (PDB ID: 1EEI).^10^ Protons were added using Schrodinger software.^14^ Protein protons directly irradiated at 2.25 ppm are depicted with orange surfaces.

Experiments conducted with differential frequencies (2.25/0.60 ppm) resulted in positive ΔSTD values for protons H4, H5, H6, and H6′ on the galactose, thus indicating an increase in relative STDs when irradiating at 2.25 ppm (Figure 4 a). In contrast, negligible ΔSTD factors were observed for H1, H2, H3, and the aromatic protons at the opposite end of the molecule. The differential epitope map of 3NPG (Figure 4 b) was found to be in perfect agreement with the published crystal structure of the complex between 3NPG and CTB (Figure 4 c),^10^ in which the galactose ring area of H4 to H6 is surrounded by the side chains of E51 and Q56. In contrast, H1, H2, H3, and the aromatic carbon atoms point away from those side chains in the binding pocket (Figure 4 c).

Finally, we conducted DEEP‐STD NMR experiments with differential D_2_O/H_2_O conditions on the 3NPG–CTB complex. The ΔSTDs of 3NPG were negligible, and no differential epitope was obtained, thus indicating that changing solvent did not significantly affect the STD pattern (Figure S4). This is in agreement with the lack of slowly exchanging polar residues in the CTB binding pocket.^10^ This suggests that when no differential epitope is obtained after a change from D_2_O to H_2_O, the protein binding pocket is likely to lack slowly exchanging polar side chains (e.g., Arg).^8^

In summary, DEEP‐STD NMR is a robust tool to get information on the nature of the amino acids surrounding the ligand in the binding site in order to assess the pharmacophore of the protein. This information is inaccessible by traditional STD NMR. Additionally, if the protein 3D structure is known, the method allows information to be gained from STD NMR on the orientation of the ligand for the first time. In comparison to the SOS‐NMR method proposed by Hajduk et al. to reveal ligand orientation,^16^ DEEP‐STD NMR does not need selective protein deuteration. We envisage that DEEP‐STD NMR in combination with classical STD NMR could become a popular approach to characterize in depth the binding of weak ligands to protein receptors. This is of great relevance to accelerate fragment‐based drug discovery efforts, an approach of increasing importance in the biopharmaceutical industry for the development of novel therapeutics.

*Dedicated to Professor Thomas Peters on the occasion of his 60th birthday*

## Conflict of interest

The authors declare no conflict of interest.

## Supporting information

As a service to our authors and readers, this journal provides supporting information supplied by the authors. Such materials are peer reviewed and may be re‐organized for online delivery, but are not copy‐edited or typeset. Technical support issues arising from supporting information (other than missing files) should be addressed to the authors.

Supplementary
