# Supplementary material for: Differential Epitope Mapping by STD NMR Spectroscopy To Reveal the Nature of Protein–Ligand Contacts
Source: Angew Chem Int Ed Engl. 2017 Oct 23;56(48):15289–93. doi: 10.1002/anie.201707682 (PMC5725711; doi:10.1002/anie.201707682)
Supplement: Supplementary file 1 — Supplementary [file ANIE-56-15289-s001.pdf]

Angewandte Chemie
Eine Zeitschrift der Gesellschaft Deutscher Chemiker

Supporting Information

Differential Epitope Mapping by STD NMR Spectroscopy to Reveal the Nature of Protein–Ligand Contacts

*Serena Monaco, Louise E. Tailford, Nathalie Juge, and Jesus Angulo**

anie_201707682_sm_miscellaneous_information.pdf

Author Contributions

S.M. Conceptualization: Supporting; Data curation: Lead; Formal analysis: Lead; Investigation: Lead; Methodology: Lead; Software: Lead; Validation: Lead; Writing—original draft: Equal; Writing—review & editing: Equal
L.T. Formal analysis: Supporting; Investigation: Supporting; Validation: Supporting; Writing—original draft: Supporting; Writing—review & editing: Supporting
N.J. Formal analysis: Supporting; Methodology: Supporting; Resources: Supporting; Writing—original draft: Equal; Writing—review & editing: Equal
J.A. Conceptualization: Lead; Formal analysis: Lead; Funding acquisition: Lead; Investigation: Lead; Methodology: Lead; Project administration: Lead; Resources: Lead; Software: Equal; Supervision: Lead; Writing—original draft: Lead; Writing—review & editing: Lead.

1. **Materials and methods……………………………………………………………………1**

2. **Selection of irradiation frequencies. Identification of residues in the binding pocket being directly irradiated (*Rg*NanH-GH33 and CTB)………..……………………2**

3. **Exchange rate of polar protons and Differential D_2O/H_2O Epitope Mapping……….4**

4. **DEEP-STD protocol…………………………………………………………………………5**

5. **STD raw and processed data………………………………………………………………7**

6. **STD NMR spectra……………………………………………………………………………9**

7. **Differential Epitope Mapping D_2O/H_2O of the CTB / 3NPG complex………………...10**

8. **CORCEMA-ST validation of Differential Epitope Mapping for *Rg*NanH-GH33…….10**

1. Materials and methods

Ligands and proteins. 3-Nitrophenyl α-D-galactopyranoside (3NPG), cholera toxin subunit B (CTB), deuterium oxide (99.9% ^{2}H), tris-(hydroxymethyl-d_3)amino-d_2-methane (Tris-d_{11}, 98% ^{2}H), disodium hydrogen phosphate (Na_2HPO_4), potassium dihydrogen phosphate (KH_2PO_4), sodium chloride (NaCl) and potassium chloride (KCl) were purchased from Sigma. 2,7-anhydro-Neu5Ac was prepared as previously described (Crost, 2016). Briefly, the compound was enzymatically synthetized by incubating 4-Methylumbelliferyl-Neu5Ac with *Rg*NanH in ammonium formate buffer (100 mM) at 37°C, pH 6.5 overnight. The reaction was terminated by the addition of an equal volume of ethanol and the precipitate removed by centrifugation (4000 *g* for 20 min). The mixture was evaporated to dryness and the 2,7-anhydro-Neu5Ac was purified by Folch partitioning.

*Rg*NanH-GH33 was expressed in *Escherichia coli* BL21 cells (New England Biolabs, Boston, USA) and purified by immobilized metal anion chromatography (IMAC) as described in Tailford et al., 2015, where this domain is denoted "*Rg*NanH NI-domain".[1]

NMR measurements. ^{1}H and ^{13}C resonance assignment for the ligands was performed on the bases of 1D 1H, 2D DQF-COSY, HSQC and NOESY experiments run on the free ligands in deuterium oxide. For STD NMR experiments, all the samples consisted of 1 mM ligand and 50 μM in binding sites (*Rg*NanH-GH33 has a single binding site per protein, whereas CTB is a pentamer with five identical binding sites). 2,7-anhydro-Neu5Ac/*Rg*NanH-GH33 samples were analyzed at 293 K and buffered in 10 mM Tris-d_{11} pH 7.8 and 100 mM NaCl; 3NPG/CTB samples were analyzed at 278 K and buffered in 10 mM PBS buffer, 137 mM NaCl and 2.7 mM KCl pH 7.4. For the experiments in light water, buffers and ligands were freeze dried and dissolved in Milli Q and the H_2O:D_2O ratio adjusted to 90:10 (10% D_2O for locking purposes). An STD pulse

sequence that included 2.5 ms and 5 ms trim pulses and a 3 ms spoil gradient was used. Saturation was achieved by applying a train of 50 ms Gaussian pulses (0.40 mW) on the f2 channel, at 0.60 ppm, 2.25 ppm or 6.55 ppm (on-resonance experiments) and 40 ppm (off-resonance experiments). The broad protein signals were removed using a 40 ms spinlock (T1ρ) filter (stddiff.3). When required, water suppression was achieved by excitation sculpting with gradient (stddiffesgp.3). All the experiments were recorded at ^{1}H frequency of 800.23 MHz on a Bruker Avance III spectrometer equipped with a 5-mm probe TXI 800 MHz H-C/N-D-05 Z BTO. STD NMR experiments with a saturation time of 0.5 s and a relaxation delay of 5 s were performed on all the samples, with 256 scans for samples in D_2O and 512 scans for samples with H_2O:D_2O 90:10. STD NMR experiments on samples containing only the ligands (blank samples) were carried out to exclude direct irradiation of ligands signals.

2. Selection of irradiation frequencies. Identification of residues in the binding pocket being directly irradiated, for proteins of known structures.

General considerations for DEEP-STD NMR studies: For proteins of known 3D structure, if their chemical shift assignments are known, directly irradiated protons can be easily identified in the binding pocket. In many cases, however, the protein chemical shifts are not available. In those cases, statistical averages of chemical shifts of the residues present in the binding pocket (from existing NMR databases) can be used. Alternatively, the 3D structure of the protein can be used to predict the chemical shifts using existing software.

In the present work, the selection of irradiation frequencies was based on predictions of chemical shifts using the available 3D structures of the proteins by using ShiftX2 (http://www.shiftx2.ca).[2] Additionally, the chemical shifts of selected protons on the proteins were also checked against average chemical shift histograms from the Biological Magnetic Resonance Data Bank (http://www.bmrb.wisc.edu/).[3] All molecular graphics were generated with Schrodinger Maestro 11 version 2016-4. Hydrogen atoms were added to the ligand and to the protein using the *Protein Preparation Wizard* and *LigPrep*, and protonated residues within 4 Å from the ligand where selected as shown Figures 2-4 of the main text.

Table S1. ^{1}H chemical shifts of amino acid residues in the binding pocket (within 4 Å from the ligand) of *Rg*NanH-GH33 (left) and CTB (right) as simulated by shiftx2.ca (ref: http://www.shiftx2.ca). Resonances within 0.4 ppm from the directly irradiated frequencies (i.e., 0.6 ppm and 6.55 ppm in the case of the differential irradiation DEEP-STD studies of the *Rg*NanH-GH33/2,7-anhydro-Neu5Ac complex, and 2.25 ppm in the case of the CTB/3NPG complex,) are highlighted in the same colour scale as in Figures 2 and 4 of the main text.

*Rg*NanH-GH33 Binding Pocket Residues				CTB Binding Pocket Residues			
**258**	**ILE**	**HA**	4.11	**12**	**TYR**	**HA**	4.63
		**HB**	2.01			**HB2**	2.73
		**HD1**	0.49			**HB3**	2.90
		**HG2**	0.98			**HD1**	7.07
		**HG12**	1.48			**HD2**	7.01
		**HG13**	1.29			**HE1**	6.8
**338**	**ILE**	**HA**	4.71			**HE2**	6.83
		**HB**	1.69	**32**	**ALA**	**H**	8.29
		**HD1**	0.60			**HA**	4.16
		**HG2**	0.84			**HB**	1.37
		**HG12**	1.25			**HB2**	1.73
		**HG13**	1.14			**HB3**	1.51
**502**	**VAL**	**HA**	4.70	**33**	**GLY**	**HA**	3.89
		**HB**	1.74			**HA2**	3.87
		**HG1**	0.70			**HA3**	3.88
		**HG2**	0.36	**51**	**GLU**	**HA**	4.35
**557**	**THR**	**HA**	3.62			**HB2**	2.02
		**HB**	4.10			**HB3**	2.08
		**HG2**	0.97			**HG2**	2.28
**525**	**TYR**	**HA**	5.98			**HG3**	2.30
		**HB2**	2.95	**56**	**GLN**	**HA**	4.05
		**HB3**	2.84			**HB2**	1.81
		**HD1**	7.02			**HB3**	1.93
		**HD2**	6.93			**HE21**	7.08
		**HE1**	6.64			**HE22**	6.87
		**HE2**	6.64			**HG2**	1.94
**677**	**TYR**	**HA**	4.66			**HG3**	2.02
		**HB2**	3.05	**90**	**ASN**	**HA**	4.83
		**HB3**	2.76			**HB2**	3.02
		**HD1**	6.82			**HB3**	2.82
		**HD2**	6.83			**HD21**	7.44
		**HE1**	6.68			**HD22**	6.97
		**HE2**	6.63	**91**	**LYS**	**HA**	4.62
**698**	**TRP**	**HA**	4.47			**HB2**	1.85
		**HB2**	3.18			**HB3**	1.72
		**HB3**	3.01			**HD2**	1.62
		**HD1**	6.63			**HD3**	1.62
		**HE1**	9.89			**HE2**	2.96
		**HE3**	7.25			**HE3**	2.94
						**HG2**	1.38
						**HG3**	1.37
						**HZ**	7.27

3. Exchange rate of polar protons and Differential D_2O/H_2O Epitope Mapping

In H_2O, the exchange rate of exchangeable polar protons on amino acid side chains of the protein in the ligand binding pocket has a strong influence on the ability of these protons (and their adjacent non-exchangeable ones) to transfer magnetization to a bound ligand. In H_2O, *slow exchanging* protons will contribute to transfer the magnetization from the binding site to the ligand. In contrast, in the case of *fast exchanging protons*, their exchange with the bulk water will be too fast for them to take part efficiently in the protein-ligand saturation transfer process. In addition, the magnetization of non-exchangeable protein protons in the binding pocket close in space to fast exchanging protein protons will be lost in the bulk water due to an efficient exchange-mediated magnetization leakage (**Figure S1**).[4]

Based on these premises, the presence of slow exchanging protons in the binding pocket will enhance the STD of ligands protons in close contact with them, if the experiment is performed in H_2O as opposed to D_2O (a red "+" in **Figure S1**). It is worth noting that the enhancement of STD is "relative"; that is, after comparison of the "binding epitopes" (relative normalized STDs) under the two conditions (H_2O and D_2O), and not by comparing the absolute STD values (see Equation-1 below).

The presence of "isolated" fast exchanging protons will not have a significant effect on the STD of the ligand protons when increasing the percentage of protonated sites over deuterated ones (in light water they will exchange fast and they will be "invisible" in terms of magnetization transfer). However, as mentioned above, the presence of fast exchanging protons near to non-exchangeable protein protons leads to leakage of the magnetization of the latter. What is more, if the latter is/are in close contact with ligand protons (X'H-H in **Figure S1**), the result will be a relative reduction of the STD of the closest ligand protons (a purple "-" in **Figure S1**). This explains the positive DEEP-STD factor D_2O/H_2O observed for the methyl group of 2,7-anhydro-Neu5Ac (>+0.75) in our study. In the X-ray structure (PDB ID: 4X4A), the methyl group of V502, which is contributing significantly to the transfer of magnetization in the STD NMR experiments (Figure 2 main text), points toward the hydroxyl group of Y525, which is known to have a very fast kinetics of exchange. [4] This induces an efficient exchange-mediated leak of magnetization from V502 to the bulk water, having the overall effect of decreasing the STD on the methyl group in H_2O (when compared to the same sample in D_2O).

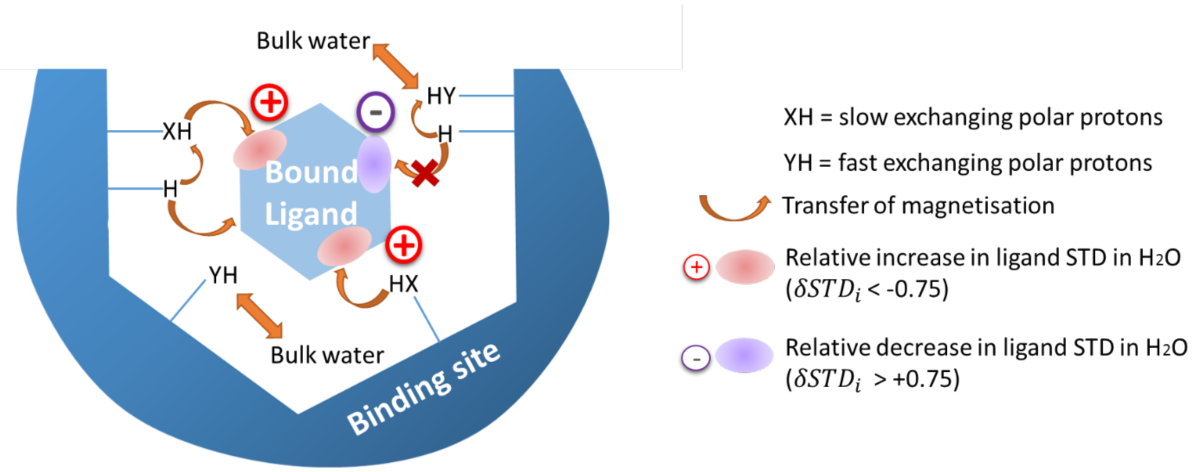

Figure S1. Cartoon representing the different pathways for saturation transfer from slow and fast exchanging polar protons of the binding pocket to the bound ligand in H_2O. **Slow exchanging protein polar protons** contribute to additional STD intensities on the ligand (**plus signs**). **Fast exchanging protons** either do not contribute, or can lead to ligand STD reduction due to exchange mediated leakage (**minus sign**). This figure can be considered as a modified version of Figure 1 in [4], adapted to explain DEEP-STD NMR experiments with differential solvent conditions (D_2O/H_2O).

4. DEEP-STD data processing protocol

The first step in the analysis of DEEP-STD NMR data consists in determining which of the two experimental conditions, experiment-1 (***exp1***) or experiment-2 (***exp2***), produced stronger STD intensities (comparing their total sum of STD values). The experiment giving rise to stronger STD intensities will be called "experiment-1". Next, the ratio of STD intensities $\left(\frac{STD\ \%_{exp1,i}}{STD\ \%_{exp2,i}}\right)$ is calculated for each proton of the ligand. These ratios report not only on differences in the epitopes but also on the different global level of protein saturation achieved in both experiments (*e.g.*, saturation on the aromatics leads to a reduced global saturation of the protein, as the number of aromatic residues is normally much lower than the number of aliphatic ones). For that reason, to obtain the differential epitope ("DEEP-STD map"), the intrinsic differences in protein saturation must be removed. This assures that the data reveal only those differences purely arising from the different types of amino acids hit by the saturating radiofrequency. To that aim, the average ratio of STDs over all protons must be calculated $\left[\frac{1}{n}\sum_i^n\left(\frac{STD\ \%_{exp1,i}}{STD\ \%_{exp2,i}}\right)\right]$. To remove the contribution from differences in saturation level, and to establish a "zero" in the scale for analysis of the differential epitope, this average factor must be subtracted to each of the individual STD ratios to find the DEEP-STD value for each proton:

$$\Delta STD_i = \frac{STD\ \%_{exp1,i}}{STD\ \%_{exp2,i}} - \frac{1}{n}\sum_i^n\left(\frac{STD\ \%_{exp1,i}}{STD\ \%_{exp2,i}}\right) \qquad \textit{Equation-1}$$

In this equation, ***exp1*** is the experiment producing the strongest set of STD intensities ($\sum \boldsymbol{STD}\ \%_{\boldsymbol{exp1,i}} > \sum \boldsymbol{STD}\ \%_{\boldsymbol{exp2,i}}$).

If all the protons are affected (by the different conditions) in a similar fashion, the ratio $\frac{STD\ \%_{exp\ 1,i}}{STD\ \%_{exp\ 2,i}}$ will be constant for all the protons and subtracting the average STD factor will give values close to 0, indicative that none of the residues contacting the ligand are sensitive to the change in conditions (as, for example, in the case of 3NPG-CTB in D_2O *vs.* H_2O in the present work). If the change in conditions is affecting the residues lining the binding pocket, we will observe positive values for protons experiencing a relative increase in STD values in **experiment 1** and negative values for protons experiencing a relative increase in STD values in **experiment 2**. Again, it is important to highlight that the increase/decrease of STD is "relative", after comparison of the binding epitopes under the two conditions, as expressed in Equation-1, and not by comparing the absolute STD values. To interpret the results from DEEP-STD NMR experiments, only the strongest ΔSTD values should be considered. Which ΔSTD values should be considered significant will depend on the sizes of the STD factors for the protein-ligand system under study. Based on the results of the systems analyzed in the present work, we experimentally determined that $\Delta\mathrm{STD}$ greater than 0.75 in magnitude were significant.

For very large multimeric protein complexes spin diffusion will play a prominent role, making the differences between different irradiations frequencies very small. Nevertheless, it might be still possible to pick them up, due to the "differential" nature of the determined epitope in the DEEP-STD NMR method. It is not possible to give an upper limit of applicability of the method as it will depend not only on the molecular weight of the receptor but also on the internal dynamics of the protein (increased internal mobility will reduce the effect of spin diffusion).

5. STD raw and processed data

Table S2. DEEP-STD NMR using *Differential Irradiation (0.6/6.55 ppm)* of the *Rg*NanH-GH33 / 2,7-anhydro-Neu5Ac complex

Proton	^{1}H δ (ppm)[a]	STD % 0.6 ppm	STD % 6.55 ppm	Ratio STD 0.60 / 6.55 ppm	ΔSTD
**CH3**	1.90	3.66	0.94	3.89	1.68
**H3a**	1.89	2.87	0.91	3.15	0.94
**H3e**	2.04	2.42	1.05	2.30	0.09
**H4**	3.82	2.84	1.38	2.06	-0.16
**H5**	3.79	2.58	0.94	2.74	0.53
**H6**	4.41	2.68	1.18	2.27	0.06
**H7**	4.30	2.61	1.29	2.02	-0.19
**H8**	3.41	1.41	1.24	1.14	-1.08
**H9**	3.46	1.03	0.89	1.16	-1.06
**H9'**	3.63	1.15	0.82	1.40	-0.81
		**Sum**	**Sum**	**STD average**	
		23.25	10.64	2.21	

[a] Spectra for assignment acquired at 293 K.

Table S3. DEEP-STD NMR using *Differential Solvent (D_2O/H_2O)* of the *Rg*NanH-GH33 / 2,7-anhydro-Neu5Ac complex

Proton[a]	STD % D_2O	STD % H_2O	Ratio STD D_2O / H_2O	ΔSTD
**CH3**	3.66	0.51	7.18	1.75
**H3a**	2.87	0.69	4.16	-1.26
**H3e**	2.42	0.59	4.10	-1.32
**H4**	2.84	0.45	6.31	0.89
**H5**	2.58	0.46	5.61	0.19
**H8**	1.41	0.27	5.22	-0.20
**H9**	1.03	0.3	3.43	-1.99
**H9'**	1.15	0.31	3.71	-1.71
	**Sum**	**Sum**	**STD average**	
	17.96	3.65	5.42	

[a] For assignment see **Table S2**.

Table S4. DEEP-STD NMR using *Differential Irradiation (2.25/0.6 ppm)* CTB / 3NPG

Proton	^{1}H δ (ppm)[a]	STD % 2.25 ppm	STD % 0.60 ppm	Ratio STD 2.25 / 0.60 ppm	ΔSTD
**H1**	5.50	6.73	3.66	1.84	-0.68
**H2**	3.73	12.51	4.95	2.53	0.01
**H3**	3.81	10.81	4.72	2.29	-0.23
**H4**	3.75	21.82	5.9	3.70	1.18
**H5**	3.73	15.21	4.6	3.31	0.78
**H6/H6'**	3.40	19.79	5.84	3.39	0.87
**H cd**	7.27	2.81	1.46	1.92	-0.60
**Hb**	7.69	5.24	2.9	1.81	-0.71
**Ha**	7.75	12.21	6.38	1.91	-0.61
		**Sum**	**Sum**	**STD average**	
		107.13	40.41	2.52	

[a] Spectra for assignment acquired at 278 K.

Table S5. DEEP-STD NMR using *Differential Solvent (D_2O/H_2O)* of the CTB / 3NPG

Proton[a]	STD % D_2O	STD % H_2O	Ratio STD D_2O / H_2O	ΔSTD
**H1**	3.66	1.4	2.61	-0.62
**H2**	4.95	1.51	3.28	0.05
**H3**	4.72	1.51	3.13	-0.10
**H4**	5.9	1.83	3.22	-0.01
**H5**	4.6	1.24	3.71	0.48
**H6/H6'**	5.84	1.6	3.65	0.42
**H cd**	1.46	0.51	2.86	-0.37
**Hb**	2.9	1.1	2.64	-0.59
**Ha**	6.38	2.02	3.16	-0.07
	**Sum**	**Sum**	**STD average**	
	40.41	12.72	3.14	

[a] For assignment see **Table S4**.

6. STD NMR spectra

6.1. STD NMR spectra with *differential irradiation*

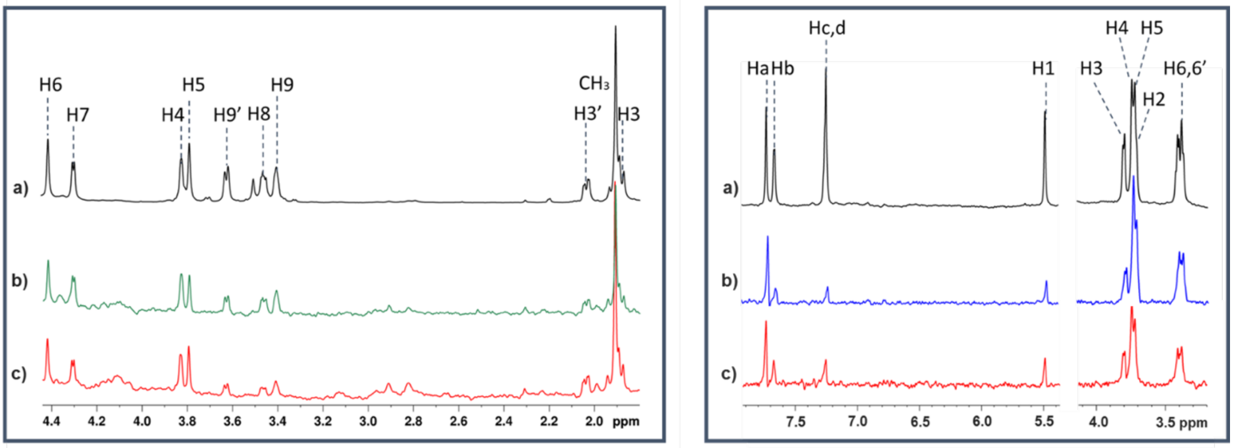

Figure S2. Left panel: 1 mM of 2,7-anhydro-Neu5Ac in the presence of 50 μM *Rg*NanH-GH33 in deuterated tris-d_{11} buffer, 293 K. **a)** Reference spectra (x 1); **b)** STD NMR spectrum with on-resonance irradiation at 6.55 ppm (x 64); **c)** STD NMR spectrum with irradiation at 0.6 ppm (x 32). **Right panel:** 1 mM of 3NPG in the presence of 10 μM CTB (50 μM binding pockets) in deuterated PBS buffer, 278 K. **a)** Reference spectra (x 1); **b)** STD NMR spectrum with on-resonance irradiation at 2.25 ppm (x 8); **c)** STD NMR spectrum with irradiation at 0.6 ppm (x 16).

6.2. STD NMR spectra with *differential solvent* conditions (D_2O/H_2O)

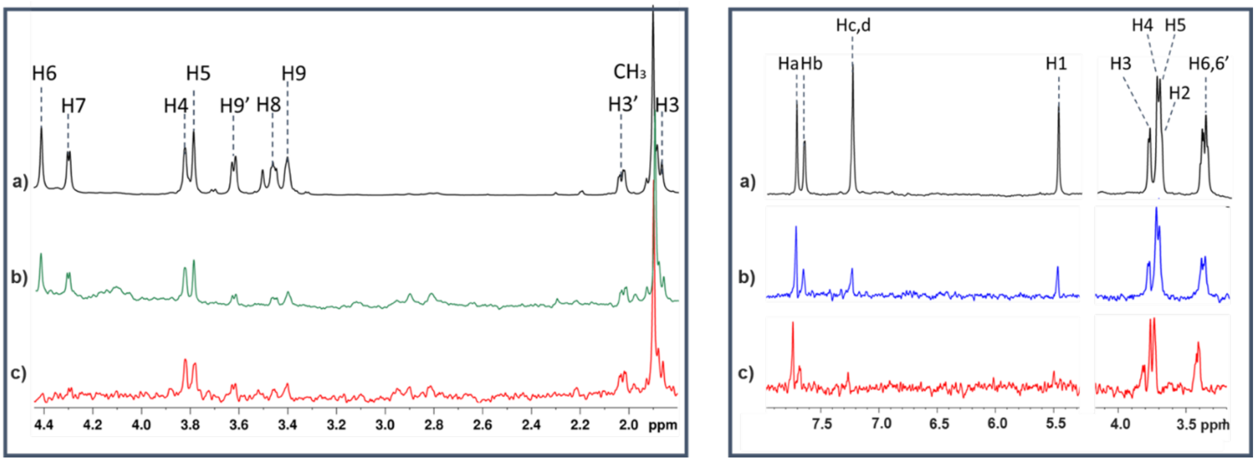

Figure S3. Left panel: 1 mM of 2,7-anhydro-Neu5Ac in the presence of 50 μM *Rg*NanH-GH33 50 uM in PBS buffer, 298 K. **a)** Reference spectra (x 1); **b)** STD NMR spectrum in D_2O (on-resonance at 0.6 ppm; off-resonance at 40 ppm) (x 32); **c)** STD NMR spectrum in H_2O:D_2O 90:10 (x 128). **Right panel:** 1 mM of 3NPG in the presence of 10 μM CTB (50 μM binding pockets), 278 K. **a)** Reference spectra (x 1); **b)** STD NMR spectrum in D_2O (on-resonance at 0.6 ppm; off-resonance at 40 ppm) (x 16); **c)** STD NMR spectrum in in H_2O:D_2O 90:10 (x 32).

7. Differential Epitope Mapping D_2O/H_2O of the CTB / 3NPG complex

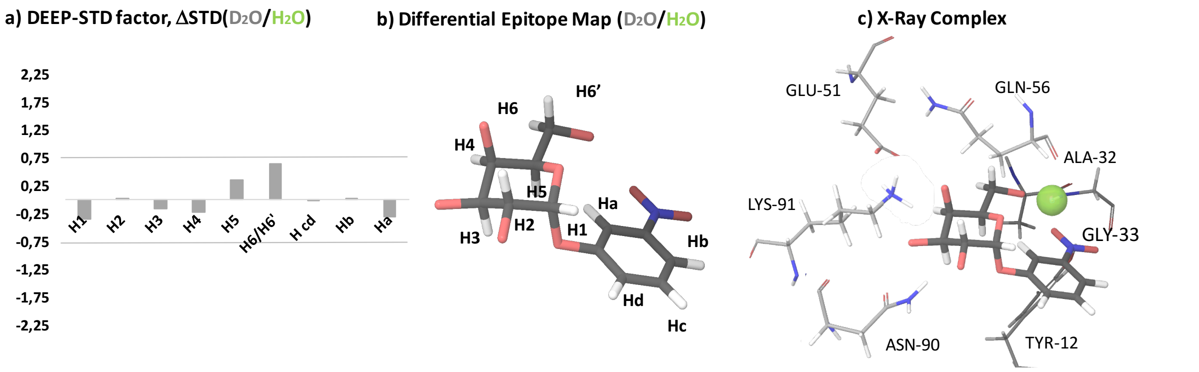

Figure S4. Differential Epitope Mapping (D_2O/H_2O) of 3NPG in complex with CTB. a) ΔSTD histogram: histograms: all the protons showed values close to 0, indicating that none of the residues in the binding pocket is affected by the change in conditions. **b) Differential Epitope**: no DEEP-STD map of the ligand was obtained. **c) Crystal structure of the complex** (PDB ID: 1EEI).[5] The slow exchangeable protons in the binding pocket are enclosed in a green surface. The ligand polar protons have been omitted.

8. CORCEMA-ST validation of Differential Epitope Mapping for *Rg*NanH-GH33

We have used theoretical full matrix relaxation calculations implemented in CORCEMA-ST[6] to validate the DEEP-STD NMR approach. CORCEMA-ST allows to predict STD intensities of a protein-ligand complex given the Cartesian co-ordinates of all the partners in the binding equilibrium (free state ligand and protein, as well as bound state complex). We tried to reproduce the differential epitope mapping at 0.5 s for the complex 2,7-anhydro-Neu5Ac with GH33, running the CORCEMA-ST calculations simulating the two approaches experimentally followed: (i) differential frequency STD (0.60 ppm/6.55ppm; Figure S5a), and (ii) differential solvent (D_2O/H_2O; Figure S5b). Even when no optimization of the parameters needed for the calculations was pursued, the theoretical results produced a pattern of positive and negative ΔSTDs (**Figure S5**) very similar to what observed experimentally (**Figure 2a** and **3a** of the main text). These theoretical results further strongly support the proposed DEEP-STD NMR approach, highlighting how depending on the architecture and chemical nature of the amino acids at the binding pocket, as well as depending on the protonation of polar side chains, the transfer of saturation to the ligand is different, and we can pick up those differences.

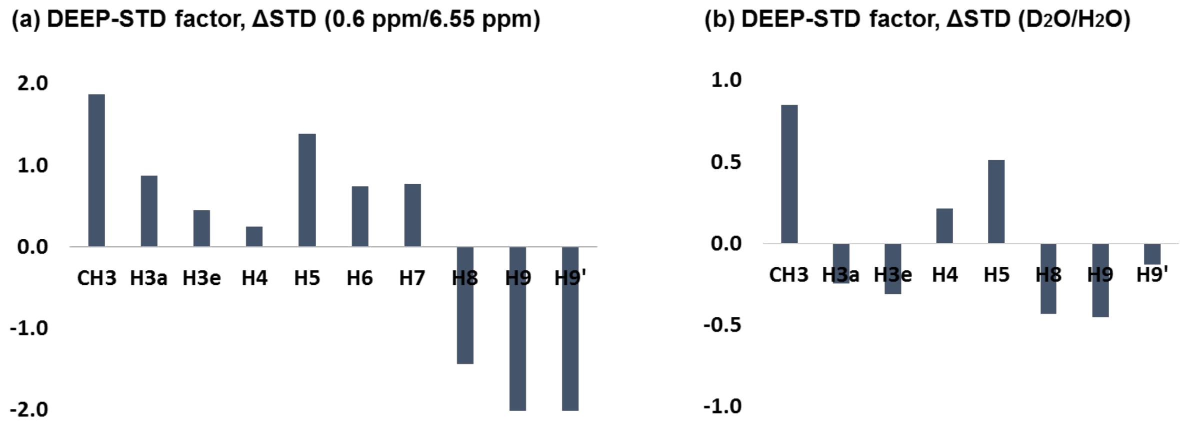

Figure S5. ΔSTD histograms for **(a)** DEEP-STD (0.6/6.55 ppm) and **(b)** DEEP-STD (D_2O/H_2O) for *Rg*NanH-GH33, as calculated using predicted STD % at 0.5 s simulated by CORCEMA-ST.

References

[1] L. E. Tailford, C. D. Owen, J. Walshaw, E. H. Crost, J. Hardy-Goddard, G. Le Gall, W. M. de Vos, G. L. Taylor, N. Juge, *Nat. Commun.* **2015**, 6.

[2] B. Han, Y. Liu, S. W. Ginzinger, D. S. Wishart, *J. Biomol. NMR* **2011**, 50, 43.

[3] E. L. Ulrich, H. Akutsu, J. F. Doreleijers, Y. Harano, Y. E. Ioannidis, J. Lin, M. Livny, S. Mading, D. Maziuk, Z. Miller, E. Nakatani, C. F. Schulte, D. E. Tolmie, R. Kent Wenger, H. Yao, J. L. Markley, Nucleic Acids Res. **2008**, 36, D402-D408.

[4] E. Liepinsh, G. Otting, *Magn. Reson. Med.* **1996**, 35, 30-42.

[5] E. K. Fan, D. A. Merritt, Z. S. Zhang, J. C. Pickens, C. Roach, M. Ahn, W. G. J. Hol, *Acta Crystallogr. D* **2001**, 57, 201-212.

[6] V. Jayalakshmi, N. Rama Krishna, *J. Magn. Reson*. **2002**, 155, 106-118.
